# Fast MS/MS acquisition without dynamic exclusion enables precise and accurate quantification of proteome by MS/MS fragment intensity

**DOI:** 10.1038/srep26392

**Published:** 2016-05-20

**Authors:** Shen Zhang, Qi Wu, Yichu Shan, Qun Zhao, Baofeng Zhao, Yejing Weng, Zhigang Sui, Lihua Zhang, Yukui Zhang

**Affiliations:** 1 Key Laboratory of Separation Science for Analytical Chemistry, National Chromatographic Research and Analysis Center, Dalian Institute of Chemical Physics, Chinese Academy of Science, Dalian, China; 2 University of Chinese Academy of Sciences, Beijing, China; 3 Center for Interactions of Proteins in Epithelial Transport, Department of Biomedicine, Aarhus University, Aarhus, Denmark

## Abstract

Most currently proteomic studies use data-dependent acquisition with dynamic exclusion to identify and quantify the peptides generated by the digestion of biological sample. Although dynamic exclusion permits more identifications and higher possibility to find low abundant proteins, stochastic and irreproducible precursor ion selection caused by dynamic exclusion limit the quantification capabilities, especially for MS/MS based quantification. This is because a peptide is usually triggered for fragmentation only once due to dynamic exclusion. Therefore the fragment ions used for quantification only reflect the peptide abundances at that given time point. Here, we propose a strategy of fast MS/MS acquisition without dynamic exclusion to enable precise and accurate quantification of proteome by MS/MS fragment intensity. The results showed comparable proteome identification efficiency compared to the traditional data-dependent acquisition with dynamic exclusion, better quantitative accuracy and reproducibility regardless of label-free based quantification or isobaric labeling based quantification. It provides us with new insights to fully explore the potential of modern mass spectrometers. This strategy was applied to the relative quantification of two human disease cell lines, showing great promises for quantitative proteomic applications.

L-iquid chromatography coupled to tandem mass spectrometry (LC-MS/MS) is an increasingly important technique for the identification and quantification of proteome and other bio-molecules^1^,^2^,^3^,^4^. For bottom up proteomics, there are mainly two kinds of widely used LC-MS/MS strategies so far. The first and most extensively used strategy is known as shotgun or discovery proteomics. Generally in this method, the MS instrument is operated in data-dependent acquisition (DDA) mode with dynamic exclusion, enabling the fragmentation of low abundant peptides^5^. In this mode, the top N abundant precursors in MS spectra are selected for fragmentation, and the resulting spectra (MS/MS spectra) are then assigned to their corresponding peptide sequences by database searching^6^. The second strategy is termed as targeted proteomics, in which the MS instrument is operated in Selected Reaction Monitoring (SRM) (also called Multiple Reaction Monitoring, MRM) mode. Within this method, a sample is queried for the presence and quantity of a limited set of peptides that have to be specified prior to data acquisition^7^. SRM does not require the explicit detection of the targeted precursors but proceeds by the acquisition, sequentially across the LC retention time domain, of predefined pairs of precursor and product ion masses, called transitions, several of which constitute a definitive assay for the detection of a peptide in a complex sample^8^. Obviously, shotgun proteomics is the best choice for discovering the maximum number of proteins from one or a few samples. It does, however, have limited quantification capabilities on large sample sets due to the “dynamic exclusion” function in DDA mode, in which the stochastic and irreproducible precursor ion selection^9^ and under-sampling^10^ will result in the fragment ions used for quantification only reflect the peptide abundances at that given time point^3^. Although the “dynamic exclusion” function can be turned off to reduce the influence of these problems, taking the limited scan speed of most mass spectrometry into consideration, it will lead to a great decrease of identified protein number^11^. In contrast, targeted proteomics is well suited for the reproducible detection and accurate quantification of sets of specific proteins in many samples as is the case in biomarker or systems biology studies^12^,^13^. At present, however, the method is limited to the measurements of a few thousands transitions per LC-MS/MS run^14^. It therefore lacks the throughput to routinely quantify large fractions of a proteome. To alleviate the limitations of both methods, strategies based on unbiased “data-independent acquisition” (DIA) have been developed^15^,^16^,^17^. However, most of the early implementations of DIA methods lost the link between the fragment ions and the precursors from which they originated, resulting in the difficulty of peptide identification. In 2012, Ruedi Aebersold and co-workers reported an alternative approach for proteome quantification termed as “SWATH MS”, which combined a DIA method with an innovative data-analysis approach based on targeted-data extraction^18^. This method permits quantification of (at least) as many compounds as those typically identified by regular shotgun proteomics with the accuracy and reproducibility of selected reaction monitoring (SRM) across many samples. Inspired by this strategy, more and more ingenious DIA based methods have been reported recently^19^,^20^,^21^,^22^. However, up to date, there are still some intractable problems remaining for this strategy. First, a library containing the proteome identification result created by DDA experiment is prerequisite to extract the target information in DIA experiment for proteome quantification, and the separation of identification and quantification not only reduces the throughput but also increases the possibility of mismatching of precursors/fragment ions in DDA spectra and fragment ions from DIA spectra. Second, the spectra of DIA data are so complicated that the accurate data extraction and analysis remain very difficult.

As mentioned above, as long as the “dynamic exclusion” function can be turned off and the scan speed is fast enough to ensure adequate PSM as for one peptide, the DDA mode has the potential of overcoming the problem of stochastic and irreproducible precursor ion selection and under-sampling, thus improving the accuracy and reproducibility of proteome quantification under the premise of not reducing the identified protein number.

In this study, we propose a strategy of fast MS/MS acquisition without dynamic exclusion to enable precise and accurate quantification of proteome by MS/MS fragment intensity. The results showed a comparable proteome identification power compared to traditional DDA method with dynamic exclusion, with better quantitative accuracy and reproducibility regardless of label-free based quantification or isobaric labeling based quantification, providing us with new insights to fully explore the potential of modern mass spectrometers. This strategy was applied to the relative quantification of human-HCC-H/L sample (cell lines MHCC97H and MHCC97L, which are HCC cells with high and low metastatic potentials, are denoted as human-HCC-H and human-HCC-L), showing great promises for applications in quantitative proteomics.

## Results and Discussion

### Protein identification

Using the same yeast peptide mixture analyzed by 1 D nanoRPLC-MS/MS under identical setting, we tested the effect of MS/MS fragmentation counts and accumulation time (top 40 with 50 ms accumulation time, top 50 with 40 ms accumulation time, top 60 with 40 ms accumulation time, top 100 with 30 ms accumulation time) on identification, as shown in Fig. S1A in Supplementary Information. Then we further tested the effect of different dynamic exclusion durations (18, 21, 24, 27 s), as shown in Fig. S1B in Supplementary Information. Then all subsequent MS data acquisition with dynamic exclusion were performed under the optimized conditions, which were top 50 MS/MS fragments with 40 ms accumulation time, and 21 s dynamic exclusion duration.

In order to test the effect of fractionation on MS data acquisition with or without dynamic exclusion, we prepared three sets of samples, which were total yeast lysis digest, total yeast lysis digest divided into two and three fractions through high pH fractionation.

**Figure 1:**
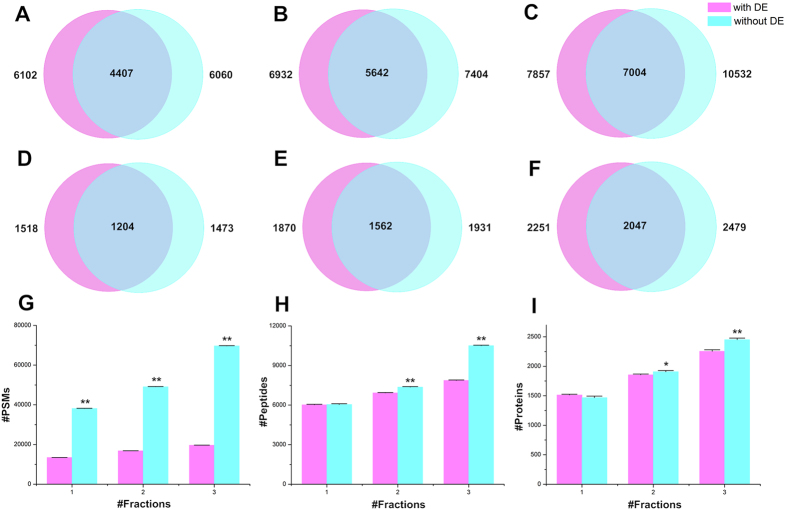
Identification in MS data acquisition with or without dynamic exclusion. Overlap of identified peptides of (**A**) total yeast lysis digest, total yeast lysis digest divided into (**B**) two and (**C**) three fractions by MS data acquisition with or without dynamic exclusion. Overlap of identified proteins of (**D**) total yeast lysis digest, total yeast lysis digest divided into (**E**) two and (**F**) three fractions by MS data acquisition with or without dynamic exclusion. Identified (**G**) PSMs, (**H**) peptides and (**I**) proteins of total yeast lysis digest with different number of fractions by MS data acquisition with or without dynamic exclusion. Data shown are average and error bars represent ±S.D., n = 3, *p < 0.05 and **p < 0.01 between with and without dynamic exclusion with 2-tailed, unpaired Student’s t-test.

As shown in Fig. 1G, compared with MS data acquisition with dynamic exclusion, the number of identified PSMs by MS data acquisition without dynamic exclusion increased dramatically no matter fractionation was performed or not. The increases in number of identified peptides (Fig. 1H) and proteins (Fig. 1I) become significant when more extensive fractionation was performed. These results suggest a more effective and comprehensive identification can be obtained by MS data acquisition without dynamic exclusion with the increase of the number of fractionation, in another word, the decrease of sample complexity in each fraction. Furthermore, as more extensive fractionation was performed, the overlap of identified peptides and proteins between two different MS data acquisition increased (Fig. 1A–F), demonstrating the credible of the results obtained by MS data acquisition without dynamic exclusion, and what is more importantly, is that our method provides a more effective way when choosing how to perform MS data acquisition, especially when fractionation is adopted.

### Label-free Quantification

For MS/MS based label-free quantification without dynamic exclusion, the ratios of peptides are calculated as following: For each MS/MS spectra, the total ion intensity is calculated by summing all of the detected fragment ions. For each peptide, the MS/MS spectra with the highest total ion intensity (spectraH) corresponding to the apex of peptide peak is chosen from all of its PSMs. Then the spectraH of same peptide in different experiments are compared to find the commonly detected fragment ions. From these commonly detected fragment ions, the top 3 ions with highest intensities are selected. The peptide ratio is calculated by comparing the summed intensities of top 3 fragment ions detected in different experiments. The protein ratio is taken as the median ratio of all peptides belonging to the same protein. For MS/MS based label-free quantification with dynamic exclusion, the intensities of each peptide is calculated by summing the intensities of all detected fragment ions in all PSMs of that peptide. The peptide ratio is calculated by comparing the summed intensities of the same peptide in different experiments and the protein ratio is taken as the median ratio of all peptides attributed to it.

**Figure 2:**
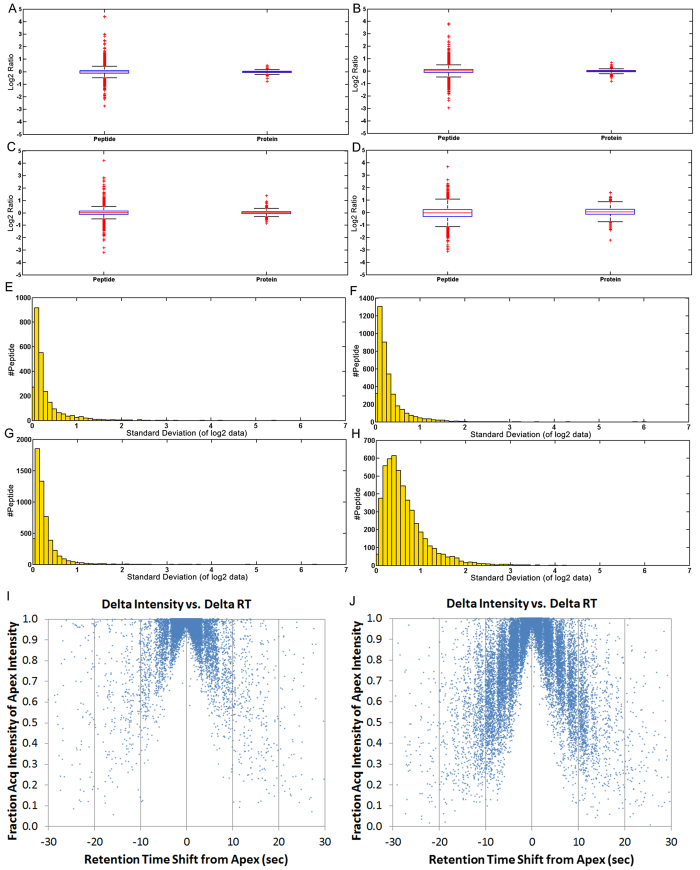
Global comparison of the effect of MS data acquisition with or without dynamic exclusion on label free based quantification by using yeast proteome (three technical replicates). Box-plot shows the measured (box and whiskers) peptide and protein ratios by (**A,B**) LFQ (XIC) or (**C,D**) label free (MS/MS) in MS data acquisition (**B,D**) with or (**A,C**) without dynamic exclusion at mixing ratios of 1:1. Histograms demonstrate the distribution of standard deviation across the log2 intensity values acquired by (E, F) LFQ (XIC) or (**G,H**) label free (MS/MS) in MS data acquisition (**F,H**) with or (**E,G**) without dynamic exclusion; For every precursor, the relationship between the best fragmentation time (represented by retention time shift from apex) and corresponding relative intensity in MS data acquisition (**I**) without or (**J**) with dynamic exclusion.

From Table 1 and Fig. 2, some conclusions can be drawn as follows. First, in Label free Quantification (LFQ) method, MS data acquisition without dynamic exclusion would decrease the identification counts (commonly quantified peptide/protein number among three technical replicates), but in MS/MS based label-free method, a slight increase of identification was observed for MS data acquisition without dynamic exclusion. Second, when the quantification accuracy and precision are taken into consideration, MS/MS based label-free method in MS data acquisition with dynamic exclusion is inferior to the other three in all aspects, and there are no obvious differences among LFQ in MS data acquisition with or without dynamic exclusion, and MS/MS based label-free in MS data acquisition without dynamic exclusion. It is worth noting that the quantitative accuracy and precision of MS/MS label-free method in MS data acquisition without dynamic exclusion is comparable to the state-of-the-art LFQ algorithm, highlighting the successful combination of MS/MS fragment intensities with MS data acquisition without dynamic exclusion. The advantage of MS/MS label-free method in MS data acquisition without dynamic exclusion compared to LFQ is that the numbers of quantifiable peptides and proteins are approximately doubled, increasing the chances of finding biologically relevant target proteins.

In order to explain the advantage of MS/MS label-free method in MS data acquisition without dynamic exclusion compared with that in MS data acquisition with dynamic exclusion, the retention time shift from the apex when the best fragmentation took place for a precursor was plotted against the fraction of that highest acquisition intensity compared to the apex intensity of that particular precursor, as shown in Fig. 2H and Fig. 2I. We can see that in MS data acquisition without dynamic exclusion, the dots are clustered around the apex, meaning that the best fragmentation took place near the apex of each precursor. On the contrary, in MS data acquisition with dynamic exclusion, the dots show a much more diverse distribution, suggesting that the best fragmentation of each precursor follows a random manner. This is because in MS data acquisition without dynamic exclusion, the same precursor was fragmented multiple times, increasing its chance to be fragmented near the apex. While in MS data acquisition with dynamic exclusion, a precursor can only be fragmented very few times, decreasing its chance to be near the apex.

Thus, the comparison of these four methods demonstrated the combination of MS data acquisition without dynamic exclusion with MS/MS fragment intensities could be an attractive alternative to state-of-the-art XIC based quantifications.

### Isobaric peptide termini labeling based quantification

We then investigated the effect of MS data acquisition with or without dynamic exclusion on quantitative precision, accuracy and reproducibility of isobarically labeled HeLa protein digests at 1:1 ratios, respectively.

**Figure 3:**
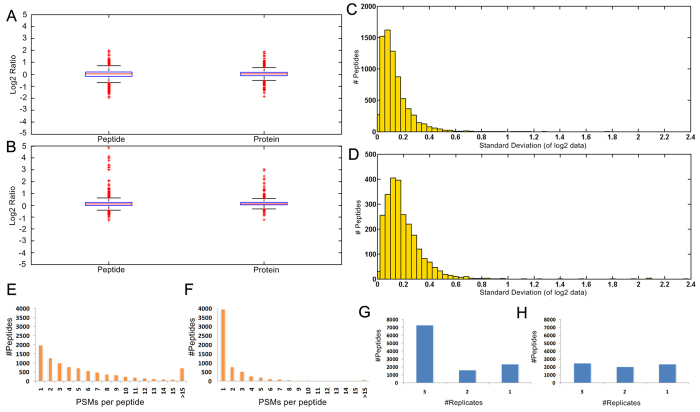
Global comparison of the effect of MS data acquisition with or without dynamic exclusion on isobaric peptide termini labeling based quantification by using HeLa proteome (three technical replicates). Box-plot shows the measured (box and whiskers) peptide and protein ratios by MS data acquisition with (**A**) or without (**B**) dynamic exclusion at mixing ratio of 1:1; Histograms demonstrate the distribution of standard deviation across the log2 intensity values acquired by MS data acquisition with (**C**) or without (**D**) dynamic exclusion; Bar graphs illustrate the number of peptides identified by different numbers of PSMs in MS data acquisition with (**F**) or without (**E**) dynamic exclusion; Bar graphs illustrate the number of peptides that could be quantified across one, two, or all three of the replicate MS data acquisition with (**G**) or without (**H**) dynamic exclusion.

As shown in Fig. 3A, in MS data acquisition without dynamic exclusion, 7271 peptides and 2375 proteins were commonly quantified among three technical replicates at median ratios of 1.025 and 1.027, with 98.8% and 98.9% of the ratios ranged from −1 to 1, and the corresponding standard deviations are 0.327 and 0.282, respectively. In MS data acquisition with dynamic exclusion, 2507 peptides and 1669 proteins were commonly quantified among three technical replicates at media ratio of 1.080 and 1.094, with 98.3% and 98.8% of the ratios ranged from −1 to 1, and the corresponding standard deviations are 0.353 and 0.298, respectively. The outliers in MS data acquisition with dynamic exclusion are biased to the heavily labeled side, and the causes have been discussed in our previous work, which might be the irreproducible precursor ion selection caused by dynamic exclusion and chromatographic shift caused by isotope effect^23^. The quantification accuracy was improved with the increase of fragmentation frequency (or the number of identified PSMs) for the peptide. As shown in Fig. 3F, in MS data acquisition with dynamic exclusion, most of the peptides could only be identified by one PSM, which reflect the irreproducible precursor ion selection. While in MS data acquisition without dynamic exclusion, most of the peptides could be identified by more than one PSM (Fig. 3E), which ameliorated the adverse effect of aforementioned two causes to some extent. The ratio distributions in MS data acquisition without dynamic exclusion also contain a few outliers, which is to be expected given the fact that there are many more peptides and proteins included in the box-plot and there are still some peptides identified by only one PSM.

Except for the obvious advantage of MS data acquisition without dynamic exclusion over with dynamic exclusion in terms of quantitative precision and accuracy, the method’s ability to perform fast MS/MS acquisition without dynamic exclusion allows this mode to quantify peptides with greater reproducibility than MS data acquisition with dynamic exclusion. Bar graphs Fig. 3G,H illustrate the number of peptides that could be quantified across one, two, or all three of the replicate experiments. MS data acquisition without dynamic exclusion was able to quantify 65.0% (7271) of the HeLa peptides across all three replicates, a significantly higher number than MS data acquisition with dynamic exclusion, which was only able to quantify 35.3% (2507) of the HeLa peptides across all three replicates. MS data acquisition without dynamic exclusion was not only able to quantify a high percentage of peptides across replicate experiments, but also the quantification values obtained for the observed peptides remain consistent among replicates. The standard deviation across the log2 intensity values acquired by the MS data acquisition with or without dynamic exclusion experiments were obtained for each peptide quantified across all three replicates, and the distribution of these standard deviations are presented in histograms Fig. 3D,E, which demonstrated the better reproducibility of MS data acquisition without dynamic exclusion than with dynamic exclusion.

Therefore the comparison between the performances of these two MS data acquisition mode demonstrated that more precise, accurate and reproducible quantitative results could be achieved by MS data acquisition without dynamic exclusion, which also indicated that this strategy might ameliorate the two potential problems of traditional isobaric labeling strategies.

### Quantitative Proteome Profiling of Human-HCC-H/L

**Figure 4:**
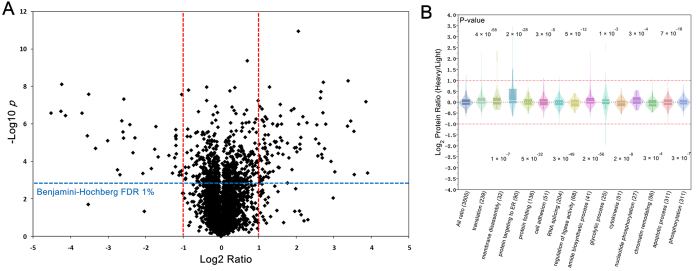
Differential analysis of human HCC H/L cell line. (**A**) Volcano plot of the global quantification of proteins in human HCC H/L cell line; (**B**) Gene ontology analysis of the differentially expressed proteins.

The isobaric peptide termini labeling based strategy was further applied to the relative quantification of human-HCC-H/L sample. The heavy-to-light ratio was obtained through PISA. Quantification ratios were then normalized based on median log2 ratio of each replicate prior to further analysis. Proteins identified and quantified in all of three replicates were delivered to Benjamini-Hochberg (BH) FDR estimations, and those passed the 1% BH-FDR threshold were retained in the volcano plot. If further using the two fold differences as the cutoff, proteins with ratio <0.5 or >2 would be considered as significant changes, as shown in Fig. 4A and Table S1 in Supplementary Dataset. Totally 4016 proteins were confidently quantified, among which 136 proteins were considered as differentially expressed with these criteria.

Gene ontology (GO) analysis suggests that almost all biological processes such as translation, protein targeting to ER and membrane disassembly are up-regulated (Fig. 4B, Table S2 in Supplementary Dataset). Among them, several ribosomal protein (Rpl34, Rpl38, Rpl21, Rps21, Rps27 *et al*.) involving in translation were quantified with high expression, and these proteins were believed to be involved, favoring the tumorigenic process, its progression and metastasis^24^. While some other biological processes such as apoptotic process, chromatin remodeling was down-regulated. In apoptotic process, three proteins were significantly low expressed. Among them, Rho GTPase-activating protein 7 (DLC1), plays a critical role in biological processes such as cell migration and proliferation. Active DLC1 increases cell migration velocity but reduces directionality^25^. PRKC apoptosis WT1 regulator protein (Pawr), capable of selectively inducing apoptosis in cancer cells, sensitizes the cells to diverse apoptotic stimuli and causes regression of tumors in animal models. The abundant quantitative results allow the in-depth biological function analysis that may be helpful for biomarker discovery and used to explain tumor invasion and metastasis.

In summary, dynamic exclusion function has proven to be a useful compromise between sampling speed and sample complexity during the past decades, when the scan speed of mass spectrometers were relatively slow. With the aid of modern ultra-high speed mass spectrometer, we demonstrate that MS data acquisition without dynamic exclusion can achieve more efficient proteome identification, better quantitative precision, accuracy and reproducibility no matter for label-free based quantification or for isobaric labeling based quantification, showing great promises in future proteomics identification and quantification.

## Experimental

### Yeast sample preparation

Yeast cells (S. cerevisiae strain S288c) grown on YPD culture medium were cultured at 37 °C for 48 h. To extract proteins from whole cells, the mixture was centrifuged at 4000 g at 4 °C for 2 min to precipitate cells. After washing with cold 1× PBS three times, 8 M urea and 1% (v/v) protease inhibitor cocktail were added to the precipitates at the ratio of 4:1 (v/m), followed by ultrasonication (Cole-Parmer, IL) for 200 s (10 s on, 10 s off, 10 runs) in an ice bath. The resulting mixture was centrifuged at 20000 g at 4 °C for 30 min, and the supernatant was collected as the soluble fraction of the extracted yeast whole cell lysate proteins. The protein concentrations were determined by a BCA assay.

The proteins from yeast dissolved in 8 M urea were respectively reduced in 10 mM DTT at 56 °C for 2 h. Subsequently, cysteines were alkylated in 25 mM IAA at room temperature for 1 h in the dark. Finally the solutions were diluted to 0.8 M urea with 50 mM sodium phosphate (pH 7.5) and trypsin was added with an enzyme to substrate ratio of 1:25 (m/m) and incubated at 37 °C overnight.

The samples were analyzed by a Triple-TOF 5600+ mass spectrometer (AB SCIEX, USA) equipped with a nano UPLC system (Eskigent, USA). The mobile phases were buffer A (97.9%H_2_O/2% ACN/0.1% FA) and buffer B (97.9% ACN/2%H_2_O/0.1% FA). The samples were loaded onto a 75 μm i.d. ×5 cm pre-column with 100% A at a flow rate of 500 nL/min, and then eluted onto a 75 μm i.d. ×20 cm analytical column. The pre-column and analytical column were both packed with C18 particles (Daiso, 5 μm, 120 Å). The flow rate was set at 300 nL/min, and the gradient was as follows: from 5 to 25% B for 80 min, then from 25% to 55% B for 24 min, and finally from 55% to 80% B for 1 min. After rinsing the columns with 80% B for 5 min, the separation system was equilibrated by 98% A for 10 min.

The Triple-TOF 5600+ was operated under a spray voltage of 2.3 kV. MS1 scan range was set from 350 to 1250 (charge state +2 to +5, cps >80). In MS data acquisition with dynamic exclusion, the MS1 accumulation time was 0.25 s, followed by 40 MS/MS scans (scan range 100–1500, accumulation time 0.05 s) using a 21 seconds exclusion window after one MS/MS event. In MS data acquisition without dynamic exclusion, the MS1 accumulation time was 0.3 s, followed by 100 MS/MS scans (scan range 100–1500, accumulation time 0.03 s).

### HeLa sample preparation

The HeLa sample was prepared according to our previous procedure^23^. Then, the isobaric labeled HeLa peptides were analyzed by a Triple-TOF 5600+ mass spectrometer (AB SCIEX, USA) equipped with a nano UPLC system (Eskigent, USA). The mobile phases were buffer A (97.9%H_2_O/2% ACN/0.1% FA) and buffer B (97.9% ACN/2%H_2_O/0.1% FA). The samples were loaded onto a 75 μm i.d. ×5 cm pre-column with 100% A at a flow rate of 3 μL/min, and then eluted onto a 75 μm i.d. ×20 cm analytical column. The pre-column and analytical column were both packed with C18 particles (Daiso, 5 μm, 120 Å). The flow rate was set at 300 nL/min, and the gradient was as follows: from 5 to 25% B for 80 min, then from 25% to 55% B for 24 min, and finally from 55% to 80% B for 1 min. After rinsing the columns with 80% B for 5 min, the separation system was equilibrated by 98% A for 10 min.

The Triple-TOF 5600+ was operated under a spray voltage of 2.3 kV. MS1 scan range was set from 350 to 1250 (charge state +2 to +5, cps >80). In MS data acquisition with dynamic exclusion, the MS1 accumulation time was 0.25 s, followed by 40 MS/MS scans (scan range 100–1500, accumulation time 0.05 s) using a 21 seconds exclusion window after one MS/MS event. In MS data acquisition without dynamic exclusion, the MS1 accumulation time was 0.3 s, followed by 100 MS/MS scans (scan range 100–1500, accumulation time 0.03 s).

### Human-HCC-H/L sample preparation

The human-HCC-H/L sample with stable isotope labeling using amino acids in cell culture (SILAC) was prepared according to our previous procedure^23^. Then, the mixture of isobarically labeled peptides from human-HCC-H/L was separated by high-pH RPLC using an Agilent 1290 Infinity LC system (Santa Clara, CA, USA) with a ZORBAX Extended-C18 LC column (2.1 × 50 mm, 1.8 μm, Agilent, USA). Buffer A (25 mM NH_4_FA in 100% H_2_O, pH = 10) and B (25 mM NH_4_FA in 90% ACN, pH = 10) were used for gradient separation. The gradient was 0–20% B (0–40 min), 20–30% B (40–50 min) and 30–80% B (50–60 min), with 20 fractions collected every 3 min. The 20 fractions were further pooled into 10 by mixing equal-interval fractions, for example, fraction 1 was mixed with fraction 11 and fraction 10 was mixed with fraction 20. The resulting 10 fractions were lyophilized in a SpeedVac, and the samples were stored at −80 °C until use.

The LC-MS/MS analysis of the mixture of isobarically labeled peptides from human-HCC-H/L in MS data acquisition without dynamic exclusion was exactly the same as that of HeLa sample.

### Data analysis

All the wiff files were converted to mgf format by AB SCIEX MS Data Converter (version 1.3 beta). As for the identification part of yeast sample, database search was performed using in-house Mascot version 2.4 to search against yeast database (downloaded from www.uniprot.org on Mar. 15th, 2013, 7,786 entries). The searching parameters were set as following: Trypsin as enzyme, 2 missed cleavage site, mass error tolerance of 0.05 Da for the precursor ions and 0.1 Da for the MS/MS fragment ions, carbamidomethylation of cystein as fixed modification, methionine oxidation as variable modification. For label free quantification based on MS, the wiff files of yeast sample were searched by Maxquant version 1.5.1.1. As for the HeLa and human HCC H/L isobaric labeling sample, all the mgf files were subjected to database search by paired ions based scoring algorithm (PISA) to obtain the quantitative results^23^. The detailed search parameters were as follows: Lys-C with full specificity and a maximum of two missed cleavages was set as enzyme. The MS and MS/MS mass tolerances were 0.05 Da and 0.1 Da, respectively. Carbamidomethylation of cysteine and Guanidination of lysine were set as fixed modification. The fasta database was the International Protein Index (IPI) human database (version 3.87). As for PISA search, the algorithm would automatically search for the counterparts of the specified variable modifications, so only three extra variable modifications needed to be specified, which were oxidation of methionine (+16 Da), light dimethylation of any N-term (+28 Da) and 13 C(6) of lysine. The b ion differences and y ion differences were set as 6.0318 Da and 6.0201 Da, respectively. All search results were filtered to 1% false discovery rate (FDR) on PSM level.

## Additional Information

**How to cite this article**: Zhang, S. *et al*. Fast MS/MS acquisition without dynamic exclusion enables precise and accurate quantification of proteome by MS/MS fragment intensity. *Sci. Rep.* **6**, 26392; doi: 10.1038/srep26392 (2016).

## Supplementary Material

Supplementary Information

Supplementary Table S1

Supplementary Table S2

## Figures and Tables

**Table 1:** Comparison between label free quantification in MS data acquisition with or without dynamic exclusion by LFQ (XIC) and label-free (MS/MS) algorithms through using yeast tryptic digest (three technical replicates).

	**LFQ (XIC)**				**Label-free (MS/MS)**			
	**DE**		**Without DE**		**DE**		**Without DE**	
	**Protein**	**Peptide**	**Protein**	**Peptide**	**Protein**	**Peptide**	**Protein**	**Peptide**
Count	696	4181	513	2563	1112	5036	1177	5447
Median ratio	0.997	1.012	0.992	0.987	1.029	0.978	1.016	1.010
S.D. of ratios	0.114	0.360	0.105	0.394	0.361	0.533	0.180	0.313
Percentage of ratios in the range 0.5–2	100%	97.3%	100%	96.8%	98.4%	93.3%	99.9%	98.4%

## References

[b1] Aebersold R. & Mann M. Mass spectrometry-based proteomics. Nature 422, 198–207 (2003). 12634793 10.1038/nature01511

[b2] Ong S. E. & Mann M. Mass spectrometry-based proteomics turns quantitative. Nat. Chem. Biol. 1, 252–262 (2005). 16408053 10.1038/nchembio736

[b3] Zhang Y. Y., Fonslow B. R., Shan B., Baek M. C. & Yates J. R. Protein Analysis by Shotgun/Bottom-up Proteomics. Chem. Rev. 113, 2343–2394 (2013). 23438204 10.1021/cr3003533 PMC3751594

[b4] Wu Q., Yuan H. M., Zhang L. H. & Zhang Y. K. Recent advances on multidimensional liquid chromatography–mass spectrometry for proteomics: From qualitative to quantitative analysis—A review. Anal. Chim. Acta. 731, 1–10 (2012). 22652259 10.1016/j.aca.2012.04.010

[b5] Domon B. & Aebersold R. Review - Mass spectrometry and protein analysis. Science 312, 212–217 (2006). 16614208 10.1126/science.1124619

[b6] Bateman N. W. . Maximizing Peptide Identification Events in Proteomic Workflows Using Data-Dependent Acquisition (DDA). Mol. Cell. Proteomics 13, 329–338 (2014). 23820513 10.1074/mcp.M112.026500 PMC3879624

[b7] Lange V., Picotti P., Domon B. & Aebersold R. Selected reaction monitoring for quantitative proteomics: a tutorial. Mol. Syst. Biol. 4, 222 (2008). 18854821 10.1038/msb.2008.61 PMC2583086

[b8] Wu C. C. . Expediting SRM Assay Development for Large-Scale Targeted Proteomics Experiments. J. Proteome Res. 13, 4479–4487 (2014). 25145539 10.1021/pr500500d PMC4184450

[b9] Zhang S. . Partially isobaric peptide termini labeling assisted proteome quantitation based on MS and MS/MS signals. J. Proteomics 114, 152–160 (2015). 25434490 10.1016/j.jprot.2014.11.014

[b10] Michalski A., Cox J. & Mann M. More than 100,000 Detectable Peptide Species Elute in Single Shotgun Proteomics Runs but the Majority is Inaccessible to Data-Dependent LC−MS/MS. J. Proteome Res. 10, 1785–1793 (2011). 21309581 10.1021/pr101060v

[b11] Zhang Y., Wen Z. H., Washburn M. P. & Florens L. Effect of Dynamic Exclusion Duration on Spectral Count Based Quantitative Proteomics. Anal. Chem. 81, 6317–6326 (2009). 19586016 10.1021/ac9004887

[b12] Cima I. . Cancer genetics-guided discovery of serum biomarker signatures for diagnosis and prognosis of prostate cancer. P. Natl. Acad. Sci. USA 108, 3342–3347 (2011). 10.1073/pnas.1013699108 PMC3044355 21300890

[b13] Chen C. . Screening of Missing Proteins in the Human Liver Proteome by Improved MRM-Approach-Based Targeted Proteomics. J. Proteome Res. 13, 1969–1978 (2014). 24597967 10.1021/pr4010986

[b14] Kiyonami R. . Increased Selectivity, Analytical Precision, and Throughput in Targeted Proteomics. Mol. Cell. Proteomics 10, doi: 10.1074/mcp.M110.002931 (2011). PMC3033677 20664071

[b15] Minogue C. E. . Multiplexed Quantification for Data-Independent Acquisition. Anal. Chem. 87, 2570–2575 (2015). 25621425 10.1021/ac503593d PMC4472008

[b16] Croft N. P. . Simultaneous Quantification of Viral Antigen Expression Kinetics Using Data-Independent (DIA) Mass Spectrometry. Mol. Cell. Proteomics 14, 1361–1372 (2015). 25755296 10.1074/mcp.M114.047373 PMC4424405

[b17] Panchaud A., Jung S., Shaffer S. A., Aitchison J. D. & Goodlett D. R. Faster, Quantitative, and Accurate Precursor Acquisition Independent From Ion Count. Anal. Chem. 83, 2250–2257 (2011). 21341720 10.1021/ac103079q PMC3217585

[b18] Gillet L. C. . Targeted Data Extraction of the MS/MS Spectra Generated by Data-independent Acquisition: A New Concept for Consistent and Accurate Proteome Analysis. Mol. Cell. Proteomics 11, doi: 10.1074/mcp.O111.016717 (2012). PMC3433915 22261725

[b19] Guo T. N. . Rapid mass spectrometric conversion of tissue biopsy samples into permanent quantitative digital proteome maps. Nat. Med. 21, 407–413 (2015). 25730263 10.1038/nm.3807 PMC4390165

[b20] Schubert O. T. . Building high-quality assay libraries for targeted analysis of SWATH MS data. Nat. Protoc. 10, 426–441 (2015). 25675208 10.1038/nprot.2015.015

[b21] Liu Y. S. . Glycoproteomic Analysis of Prostate Cancer Tissues by SWATH Mass Spectrometry Discovers N-acylethanolamine Acid Amidase and Protein Tyrosine Kinase 7 as Signatures for Tumor Aggressiveness. Mol. Cell. Proteomics 13, 1753–1768 (2014). 24741114 10.1074/mcp.M114.038273 PMC4083113

[b22] Tsou C. C. . DIA-Umpire: comprehensive computational framework for data-independent acquisition proteomics. Nat. Methods 12, 258–264 (2015). 25599550 10.1038/nmeth.3255 PMC4399776

[b23] Zhang S. . A paired ions scoring algorithm based on Morpheus for simultaneous identification and quantification of proteome samples prepared by isobaric peptide termini labeling strategies. Proteomics 15, 1781–1788 (2015). 25643849 10.1002/pmic.201400262

[b24] de las Heras-Rubio A., Perucho L., Paciucci R., Vilardell J. & Lleonart M. E. Ribosomal proteins as novel players in tumorigenesis. Cancer Metastasis Rev. 33, 115–141 (2013). 24375388 10.1007/s10555-013-9460-6

[b25] Kim T. Y. . Effects of Structure of Rho GTPase-activating Protein DLC-1 on Cell Morphology and Migration. J. Biol. Chem. 283, 32762–32770 (2008). 18786931 10.1074/jbc.M800617200 PMC2583296

