# Supplementary material for: Fast MS/MS acquisition without dynamic exclusion enables precise and accurate quantification of proteome by MS/MS fragment intensity
Source: Sci Rep. 2016 May 20;6:26392. doi: 10.1038/srep26392 (PMC4873735; doi:10.1038/srep26392)
Supplement: Supplementary Information [file srep26392-s1.doc]

**Supplementary Information for**

**Fast MS/MS acquisition without dynamic exclusion enables precise and accurate quantification of proteome by MS/MS fragment intensity**

Shen Zhang, Qi Wu, Yichu Shan, Qun Zhao, Baofeng Zhao, Yejing Weng, Zhigang Sui, Lihua Zhang, and Yukui Zhang

**
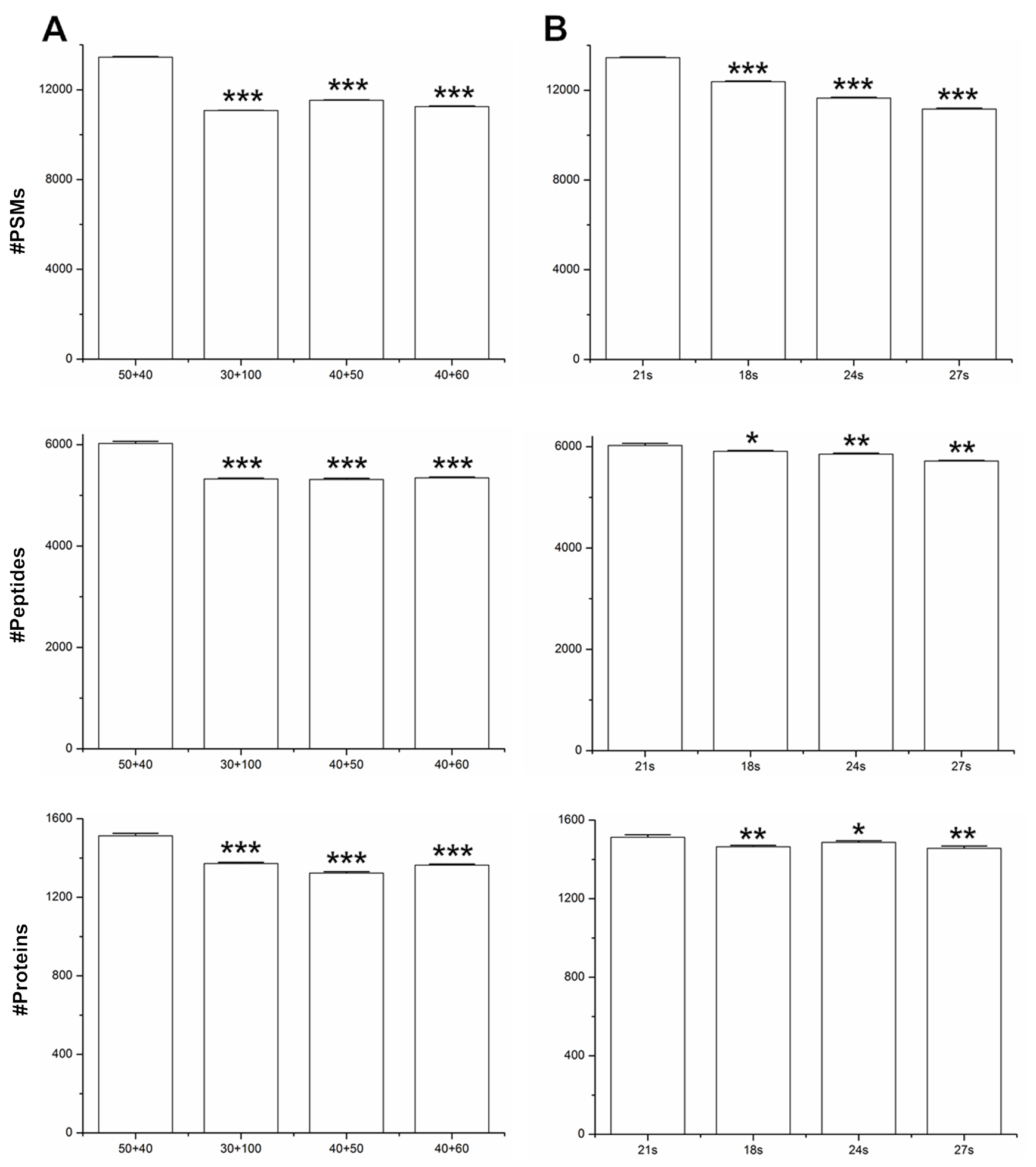
**

**Figure S1.** The effect of different MS/MS scan number, different MS/MS accumulation time (A) and different dynamic exclusion time (B) on identification. Data shown are average and error bars represent ±S.D., n = 3, **p* < 0.05 , ***p* < 0.01 and ****p* < 0.001 with 2-tailed, unpaired Student’s t-test. All comparisons are against the first bar in each figure.
